# Determination of tissue contributions to the circulating lipid pool in cold exposure via systematic assessment of lipid profiles

**DOI:** 10.1016/j.jlr.2022.100197

**Published:** 2022-03-15

**Authors:** Raghav Jain, Gina Wade, Irene Ong, Bhagirath Chaurasia, Judith Simcox

**Affiliations:** 1 Department of Biochemistry, University of Wisconsin-Madison, Wisconsin, USA; 2 Department of Biostatistics and Medical Informatics, University of Wisconsin School of Medicine and Public Health, University of Wisconsin-Madison, Wisconsin, USA; 3 Division of Endocrinology, Department of Internal Medicine, Carver College of Medicine, Fraternal Order of Eagles Diabetes Research Center, University of Iowa, Iowa, USA

**Keywords:** lipidomics, ceramides, cold exposure, MS, acylcarnitines, metabolism, thermogenesis, brown adipose tissue, regression analysis, computational tool, ACar, acylcarnitine, BAT, brown adipose tissue, Cer, ceramide, eWAT, epidydimal WAT, GSM, gastrocnemius skeletal muscle, id, identification, IPA, isopropyl alcohol, IS, internal standard, iWAT, inguinal WAT, MeOH, methanol, MTBE, methyl tert-butyl ether, PC, phosphatidylcholine, TG, triglyceride, WAT, white adipose tissue

## Abstract

Plasma lipid levels are altered in chronic conditions such as type 2 diabetes and cardiovascular disease as well as during acute stresses such as fasting and cold exposure. Advances in MS-based lipidomics have uncovered a complex plasma lipidome of more than 500 lipids that serve functional roles, including as energy substrates and signaling molecules. This plasma lipid pool is maintained through regulation of tissue production, secretion, and uptake. A major challenge in understanding the lipidome complexity is establishing the tissues of origin and uptake for various plasma lipids, which is valuable for determining lipid functions. Using cold exposure as an acute stress, we performed global lipidomics on plasma and in nine tissues that may contribute to the circulating lipid pool. We found that numerous species of plasma acylcarnitines (ACars) and ceramides (Cers) were significantly altered upon cold exposure. Through computational assessment, we identified the liver and brown adipose tissue as major contributors and consumers of circulating ACars, in agreement with our previous work. We further identified the kidney and intestine as novel contributors to the circulating ACar pool and validated these findings with gene expression analysis. Regression analysis also identified that the brown adipose tissue and kidney are interactors with the plasma Cer pool. Taken together, these studies provide an adaptable computational tool to assess tissue contribution to the plasma lipid pool. Our findings have further implications in understanding the function of plasma ACars and Cers, which are elevated in metabolic diseases.

## Graphical abstract

There are over 500 identified lipids in circulating plasma, many without known origin or function. Using untargeted lipidomics on plasma and nine other tissues of cold-exposed mice, we identified novel regulation of circulating acylcarnitines through the kidney and intestine and a multiorgan system that regulates plasma ceramides. Our findings offer new targets for the study and functional characterization of circulating lipids in acute cold exposure and a computational resource for other investigators to explore multitissue lipidome remodeling during cold exposure.

**Figure:**
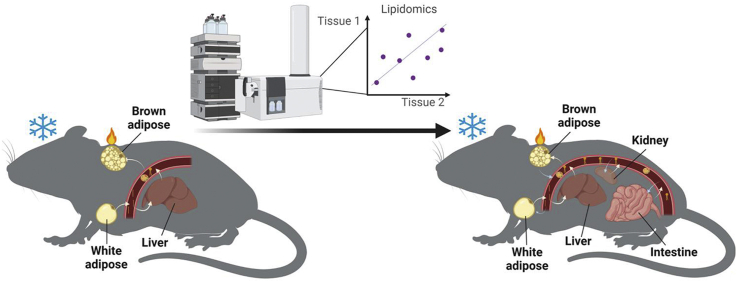

Plasma lipids reflect metabolic stress and disease. The advent of MS-based lipidomics has expanded the use of plasma lipids as diagnostic markers including newborn screens for inborn errors of metabolism to assess specific plasma acylcarnitines (ACars) (1, 2). These technological advances have also determined that there are more than 500 identified lipids in the human plasma lipidome (3). Plasma lipids have diverse structure with variations in head group, backbone, and acyl chain configuration. Despite the extended knowledge that plasma lipids can indicate metabolic abnormalities and disease, little is known about the functional role, regulation, or tissue of origin for the majority of lipids found in the circulation.

The abundance of plasma lipids is dynamically regulated to respond to nutrient availability. In fasting, there is an increase in plasma FFAs, which is driven by white adipose tissue (WAT) lipolysis, signaling a shift from glucose to lipid catabolism. In the fed state, a rise in dietary triglycerides (TGs), packed in chylomicrons, coincides with glucose-stimulated insulin secretion to induce adipocyte lipid storage. Beyond nutrient availability, factors such as age, diet, sex, and genetic background regulate lipid uptake and secretion into plasma, demonstrating the tight regulation of the plasma lipidome (4, 5). In addition to serving as energy substrates, plasma lipids have been recognized as endocrine signals with novel classes of lipids such as fatty acid esters of hydroxy fatty acids regulating insulin sensitivity (6). More work is needed to understand how the physiological context affects the complex fates of plasma lipids.

Efforts to determine the regulation and function of lipids and lipidome remodeling have utilized acute stresses, such as fasting, circadian rhythm disruption, and cold exposure to rapidly alter tissue lipid profiles (7, 8, 9, 10). Cold exposure is an energy-demanding selective pressure that requires rapid mobilization of lipids as a fuel and signal to activate mitochondrial oxidation (11). By using the stress of cold exposure, interorgan lipid regulatory pathways have been identified including the regulation of adipocyte lipolysis by hepatic insulin signaling and the regulation of bile acid production and secretion (12, 13). Our previous work utilized cold exposure to determine that plasma ACars are produced through multitissue lipid processing. In this system, cold activates the release of FFAs from the WAT for uptake by the liver. This FFA uptake results in transcriptional activation of liver ACar production and export into the plasma, where ACars are taken up by the brown adipose tissue (BAT) to serve as a fuel source for thermogenesis (9). These studies were the first to identify and track the complexity of lipid processing through multiple tissues and to characterize a functional role for ACars in thermogenesis. The results further highlight the utility of cold exposure to delineate the regulation and function of plasma lipids.

We built upon our previous work to better understand the contribution of various tissues to the plasma lipidome in acute cold exposure. After optimizing the lipid extraction for a broad range of tissues, we performed global lipidomics on the plasma, liver, BAT, inguinal WAT (iWAT), epidydimal WAT (eWAT), kidney, intestine, lung, heart, and gastrocnemius skeletal muscle (GSM) of mice kept at room temperature or exposed to cold (4°C) for 6 h. We observed the greatest cold-induced lipid changes in the liver, BAT, and plasma, with the most dynamic lipid classes being ACars, TGs, and sphingolipids. Through correlation analysis, we were able to replicate previous observations that ACars from the plasma are produced in the liver and catabolized by the BAT. Regression analysis identified previously unknown regulation of these plasma ACars by the intestine and kidney. Gene expression analysis confirmed a likely role for the intestine as a tissue of uptake and the kidney as a site of production. We extended this analysis and found that ceramides (Cers), a type of signaling sphingolipid, are increased in the plasma with acute cold exposure. Regression analysis showed that the BAT is a major regulator of the plasma Cer pool. These studies demonstrate that numerous tissues contribute to the circulating lipid pool of ACars and Cers and determined that computational assessment was able to identify novel tissue contribution. Future work will be needed to establish the functional role of plasma Cer species in acute cold exposure.

## Materials and methods

### Mouse husbandry

All animal procedures were approved by the IACUC at the University of Wisconsin-Madison. Mice were housed at room temperature (21–23°C) and 40–70% humidity using a 12 h light/12 h dark cycle. Mice were fed a standard chow diet (LabDiet, Formulab Diet 5008) and given ad libitum access to food and water. Male C57BL6J mice aged 12–14 weeks were used in all experiments. Mice were purchased from Jackson Laboratories.

### Cold tolerance test

Beginning at zeitgeber time 3, mice were placed at either 22°C (room temperature) or 4°C and 30% humidity (cold exposure) for 6 h. During the duration of the cold tolerance test, mice were singly housed with no food or bedding but free access to water. Animals were evaluated hourly for abnormal physiological changes.

### Tissue collection and processing

Mice were anesthetized by isoflurane and euthanized by cervical dislocation. Blood samples were collected by cardiac puncture in tubes containing 0.5 ml 129 mM buffered sodium citrate (Covidien) for plasma extraction. Excised tissue was washed in sterile PBS prior to sectioning and snap freezing.

### Reagents and standards

All reagents were LCMS grade or better. Chloroform, methanol (MeOH), and isopropyl alcohol (IPA) were purchased from Sigma Aldrich (catalog nos.: 132950, 1060351000, and 34863, respectively), ethyl acetate from Honeywell (catalog no.: UN1173), and methyl tert-butyl ether (MTBE) and water from Thermo Fisher Scientific (catalog nos.: E1274 and 51140, respectively). Butylated hydroxytoluene (catalog no.: B1378; Sigma-Aldrich) was added as an antioxidant to MeOH or IPA at a concentration of 0.02% (w/v) unless otherwise stated. Avanti SPLASH Lipidomix (catalog no.: 330707-1EA) and oleoyl-carnitine_d3_ (catalog no.: 26578; Cayman Chemical Company) were used as internal standards (ISs).

### Lipid extractions

For all methods, 10 μl of SPLASH mix and 300 pmol oleoyl-carnitine_d3_ was added per sample as IS, and processing blanks without IS were run for each method. Tissue was homogenized in ceramic 1.4 mm bead tubes (catalog no.: 13113-50) using the Qiagen TissueLyzer II (catalog no.: 9244420) for two to six cycles using chilled (4°C) blocks (supplemental Methods). Homogenized samples were centrifuged at 4°C for 10 min at 16,000 *g* to induce phase separation and/or pellet extracted protein prior to organic solvent transfer into new 1.5 ml microcentrifuge tubes. Extractions were done on ice using cold solvents. Lipid extracts were dried in a SpeedVac and resuspended in 100% MeOH without butylated hydroxytoluene for all tissues except iWAT and eWAT, which were resuspended in 100% IPA for better solubility of TGs. Samples were stored at −20°C for no more than 1 week and freeze-thawed <3 times prior to analysis.

#### Folch method

Tissue was homogenized in 2:1 CHCl_3_:MeOH containing IS at a volume of 100 μl per 5 mg tissue or 20 μl plasma, and 150 μl water was added to induce phase separation (14, 15). Tubes were inverted several times, centrifuged, and the top aqueous layer was transferred to a new 1.2 ml tube. About 500 μl of 2:1 solution was added to the aqueous layer for re-extraction, the tube was inverted, centrifuged, and the top aqueous layer was aspirated. A glass pipette was used to pool the organic layers in a 1.5 ml tube prior to drying.

#### MTBE method

Tissue was homogenized in 300 μl of MeOH containing IS. About 500 μl of MTBE was added, followed by 300 μl water to induce phase separation. Tubes were inverted to mix, centrifuged, and the top organic layer was extracted into a new 1.5 ml tube. Remaining aqueous phase was re-extracted with 500 μl MTBE and pooled with the first extract for drying (16).

#### Acidified MTBE method

The MTBE method was repeated with a slight modification. After extracting the initial organic layer, 100 μl of MeOH with 1% formic acid (v/v) was used to acidify the aqueous layer prior to re-extraction with 500 μl of MTBE (15).

#### IPA method

Tissue was homogenized in 500 μl of a 3:1:6 IPA:H_2_O:ethyl acetate solution containing IS (17, 18). Following homogenization, samples were placed in −20^°^C for 10 min to precipitate protein, centrifuged, and the entire solvent layer was extracted into a new tube. If tissue particles were still visible, samples were re-spun, and supernatant was transferred to a new tube for drying.

### LC/MS parameters

Extracts were separated on an Agilent 1260 Infinity II UHPLC system using an Acquity BEH C18 column (Waters 186009453; 1.7 μm 2.1 × 100 mm) maintained at 50^°^C with VanGuard BEH C18 precolumn (Waters 18003975). The chromatography gradient comprised of mobile phase A containing ACN:H_2_O (60:40), 10 mM ammonium formate, and 0.1% formic acid and mobile phase B containing 9:1:90 ACN/H_2_O/IPA, 10 mM ammonium formate, and 0.1% formic acid run at a flow rate of 0.5 ml/min. The mobile phase gradient for positive and negative ionization modes began with 15% mobile phase B increased to 30% over 2.40 min, then increased to 48% until 3 min, next to 82% at 13.2 min, then increased to 99% from 13.2 to 13.8 min, and held from 13.8 to 15.4 min before re-equilibration to 15%, held until 20 min.

The UHPLC system was connected to an Agilent 6546 Q-TOF MS dual AJS ESI mass spectrometer. For positive mode, the gas temperature was kept at 250^°^C, gas flow at 12 l/min, nebulizer at 35 psig, sheath gas temperature at 300^°^C, and sheath gas flow at 11 l/min. Negative-mode gas temperature and flow were the same while the nebulizer was kept at 30 psig, sheath gas temperature at 375^°^C, and sheath gas flow at 12 l/min. The VCap voltage was set at 4,000 V, skimmer at 75 V, fragmentor at 190 V, and Octopole radiofrequency peak at 750 V for both ionizations. Samples were injected in a random order and scanned between *m/z* 100 and 1,500. Reference masses used for positive mode were *m/z* 121.05 and 922.00. For negative mode, they were *m/z* 112.98 and 966.00. Tandem MS was performed at a fixed collision energy of 25 V. The injection volume was 3 μl for positive mode and 5 μl for negative mode. All materials, company, and company identifier are found in the key resource table in the supplemental data.

### Gene expression

RNA was isolated from liver, BAT, GSM, heart, kidney, and intestine using TRIzol reagent (Invitrogen). Samples were homogenized with a TissueLyzer II (Qiagen). Reverse transcription was performed with High-Capacity cDNA Reverse Transcription Kit (Thermo Fisher Scientific). Quantification of gene expression was performed with PowerUp SYBR Green 2x Master Mix (Thermo Fisher Scientific) on an Applied Biosystems QuantStudio 5 Real-Time PCR System, 384 wells. Relative expression was extrapolated from a standard curve for each primer pair and normalized to expression of the housekeeping gene Rps3. All primers are found in the RT-PCR primer table in the supplemental data.

### Data processing

Raw data were collected in .d format, and MS/MS data were analyzed using Agilent MassHunter Qualitative Analysis and LipidAnnotator for lipid identification (19). Lipids identified in LipidAnnotator were exported to PCDL format to create individual comprehensive libraries for each tissue. Identifications for select lipids from different class were checked for accuracy through retention time correlations with lipids of the same class and fragmentation pattern assessment. Data to compare the lipidome of cold versus room temperature mice were collected in MS1 and imported into Agilent Profinder for lipid identification and peak integration using the tissue-specific libraries. Data were exported to .csv files, and in-house R scripts were used for normalization to ISs and starting tissue amount (R Core Team, *R*, version 4.0.2).

Lipid annotations were further screened for redundancies through criteria based on analysis of raw chromatograms. The reasons for these redundancies included multiple adducts of the same lipid, slight retention time differences corresponding to the same lipid peak, and in-source fragmentation. As these redundant annotations were also present for lipid standards, we developed additional filtering criteria to obtain high-confidence unique lipid identifications. For positive ionization, individual identifications for the same lipid were filtered by dropping any duplicates if retention time difference for identication <0.10 min or if the lower intensity annotation was <25% of the more abundant identification. Cutoffs were determined by evaluating duplicate annotations for ISs. To determine total unique identifications, positive and negative mode data were compared for identical lipids annotated in both modes, and the lower abundance identification was dropped. All raw data files have been deposited to MetaboLights (#), and *R* code is available on Github (URL). Any other files are available upon request.

### Statistics

Statistical analyses of lipidomic data were performed in R using *tidyverse* (20). Lipid data were normalized to the appropriate IS and reported in pmol lipid/mg tissue except for plasma, which is in units pmol lipid/ml plasma. Principal component analyses were conducted using the *factoextra* package. Mean ± SD was reported, and *P* values less than 0.05 were considered significant unless otherwise stated. An *n =* 6 mice was used for all treatment groups. Mice were considered outliers if lipid values were >2 SD in a given comparison. To determine which lipids were changed in cold for each tissue, the log_2_(fold change) in cold versus room temperature was plotted against the −log_10_(*t*-test *P* value) for each lipid. *P* values were corrected for false discovery and considered significant if *q* <0.30 as this was a discovery-based analysis. Pearson correlations were used to determine relationships between lipid species in different tissues after outlier removal.

For regressions, lipid data were log_2_ transformed, mean centered, and scaled to the SD. Each lipid identified in solid tissue was then individually regressed against the plasma lipid of interest with temperature as a covariate using the following model:

$${\text{Lipid\_Y}}_{\text{Plasma}}={\text{β}}_{\text{0}}+{\text{β}}_{1}*\text{Temperature}+{\text{β}}_{2}*{\text{Lipid\_X}}_{\text{T}}$$

where Lipid_Y_Plasma_ was the plasma lipid of interest, β_0_ was the *y*-intercept (effectively 0 because of mean centering), temperature was a binomial where 0 was cold and 1 was room temperature, and Lipid_X was a given lipid from tissue (T) being tested as a potential predictor. Lipids were considered significantly predictive if both the temperature and lipid terms had a *P* < 0.05 as this was indicative of predictive power after accounting for the independent effect of temperature. We also tested regression models with inclusion of an interaction between lipid and temperature, but the term was not significant in our regressions. Further information on packages used throughout the analyses can be found in the supplemental Methods section.

## Results

### Optimization of lipid extraction methodology is required for multitissue analysis

**Fig. 1:**
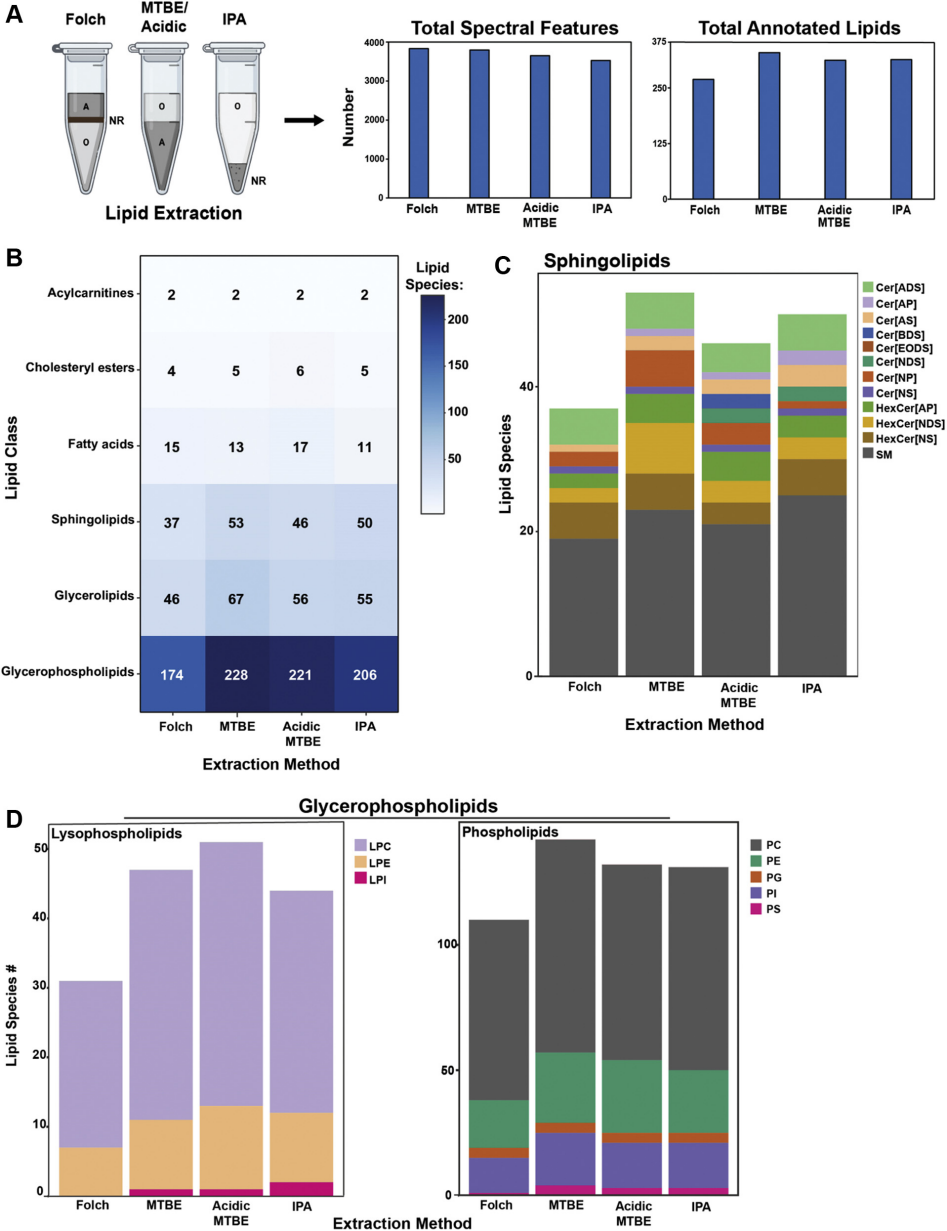
Extraction methods enrich for different lipid classes. A: Comparison of phase separation between two-phase Folch and MTBE and single-phase IPA extractions, where A = aqueous, O = organic, NR = nonrecoverable (protein). Total spectral features and total lipid identifications are shown. Lipids were identified in LipidAnnotator following LC/QTOF-MS/MS data collection on pooled plasma from male C57BL6J mice (*n* = 3). B: Heat map showing the distribution of lipid identification between the four extractions. C: Sphingolipid identification between the four extractions with distribution across subclasses shown by bar color. D: Distribution of glycerophospholipids split by lysophospholipids and phospholipids between the four methods. Bar color indicates further breakdown into subclasses. Data represent summed features and annotations from positive and negative ionization modes after manual curation.

The method of tissue homogenization and organic extraction affects lipid recovery (15). To evaluate the impact of extraction method on lipid recovery in various tissues, we tested four different solvent extractions including Folch, MTBE, acidified MTBE (acidic), and isopropanol (IPA). The Folch, MTBE, and acidic methods allow lipid recovery by separation of an aqueous and lipid-containing organic phase (Fig. 1A). In contrast, the single-phase IPA method relies on precipitation of a nonrecoverable fraction with lipids suspended in solution. We pooled lipid extracts for plasma from three mice and performed LC-MS/MS analysis to determine total spectral features and lipid identifications in positive and negative ionizations using LipidAnnotator. Computational filtering was applied to ensure unique and high-confidence lipid annotation. The Folch method yielded the highest number of total spectral features (3,912) but fewest lipid identifications (278 lipids) of all methods. The MTBE method captured the highest number of identifications (368 lipids) for plasma (Fig. 1B). Because lipid extractions perform differently based on tissue type (21), we repeated the extractions for the GSM and liver (supplemental Fig. S1A). The Folch method resulted in the highest number of features, but the IPA extraction provided the most lipid identifications for both liver (638) and GSM (434). These results indicated that the IPA method performed well for solid tissue extractions, but the MTBE method had slightly higher lipid annotation for plasma.

To determine if differences in identified lipids were driven by overall lipid recovery or because of preferential extraction of lipids between methods, we compared the number of identifications for each lipid class across the extractions. In plasma, the Folch method had the lowest identifications of all the main lipid classes, including sphingolipids, glycerolipids, and glycerophospholipids (Fig. 1B). In contrast, the MTBE method had the most identifications but was only slightly higher than the IPA method with ∼40 unique lipids varying between the groups; the major contributing lipid class to these differences was glycerophospholipids. We further delved into these differences by looking at the breakdown of lipid subclasses within each major class. The greatest variance was observed in sphingolipids. We noticed that although the total number of sphingolipids was similar between MTBE and IPA extractions, the distribution of subclasses indicated greater hexosylceramide recovery in MTBE (Fig. 1C). This was offset by more sphingomyelin species in the IPA extraction as well as the identification of ceramide nonhydroxy fatty acid dihydrosphingosine species, which were not detected in MTBE. Phosphatidylcholine (PC) species, the most abundant phospholipid in mammals, were the main lipids enriched in MTBE compared with IPA extraction (Fig. 1D). No lysophosphatidylinositols were detected in the Folch extractions, and there was a general decrease in lysophospholipids detected in Folch compared with all other methods. These findings are relevant in the context of targeted studies in plasma, which may prioritize the detection of certain lipids over others.

The IPA method had consistent and comprehensive annotation for all lipid classes and subclasses when compared with the other extractions in the liver (supplemental Fig. S1). This was also true in GSM, except for sphingolipids, which were enriched in the Folch method compared with all others (supplemental Fig. S1). In GSM, the increased identification of sphingolipids in the Folch method was due to higher detection of Cer species (>27–32 species) but fewer sphingomyelins (<4–7 species). Interestingly, we saw nearly double the number of lysophospholipid species in acidic than IPA, Folch, or MTBE methods in GSM but not in liver or plasma. This is potentially caused by increased hydrolysis of PC and phosphatidylethanolamine species, which are highly enriched in GSM, since acidic environments can promote phospholipid breakdown (22). We concluded that the IPA method was best suited for the extraction of different tissue and the recovery of diverse lipid species.

### Individual tissues have distinct cold-induced lipid remodeling

Acute cold exposure is a metabolic stress that induces rapid lipid remodeling across multiple tissues (11, 23). This lipid remodeling is driven by increased adipose tissue lipolysis, which then leads to hepatic steatosis and elevated plasma FFAs and TGs (12, 24, 25). These lipolysis-induced changes are similar to phenotypes of fasted and high-fat diet-fed mice but occur on a faster time scale with major changes in 5 h of cold exposure, mimicking 24 h of fasting (9, 24, 26, 27, 28). We have previously employed the model of cold exposure to demonstrate a functional role for plasma ACars as fuel for BAT thermogenesis (9). This finding highlights the plasticity of lipids in response to physiologic stress and the potential of rapid and cold-induced lipid remodeling to provide insight into lipid functions. The observations also unearthed the complexity of lipid processing contributing to the plasma lipid pool since ACar production is induced by WAT lipolysis of TGs into FFAs, which are then processed into ACars by the liver, secreted into circulation, and taken up by BAT (9). These studies were limited by a focused exploration of known contributors to the plasma ACar pool. More work is needed to understand the tissue of origin for other circulating lipid as well as the functional contribution of other tissues to thermogenesis.

**Fig. 2:**
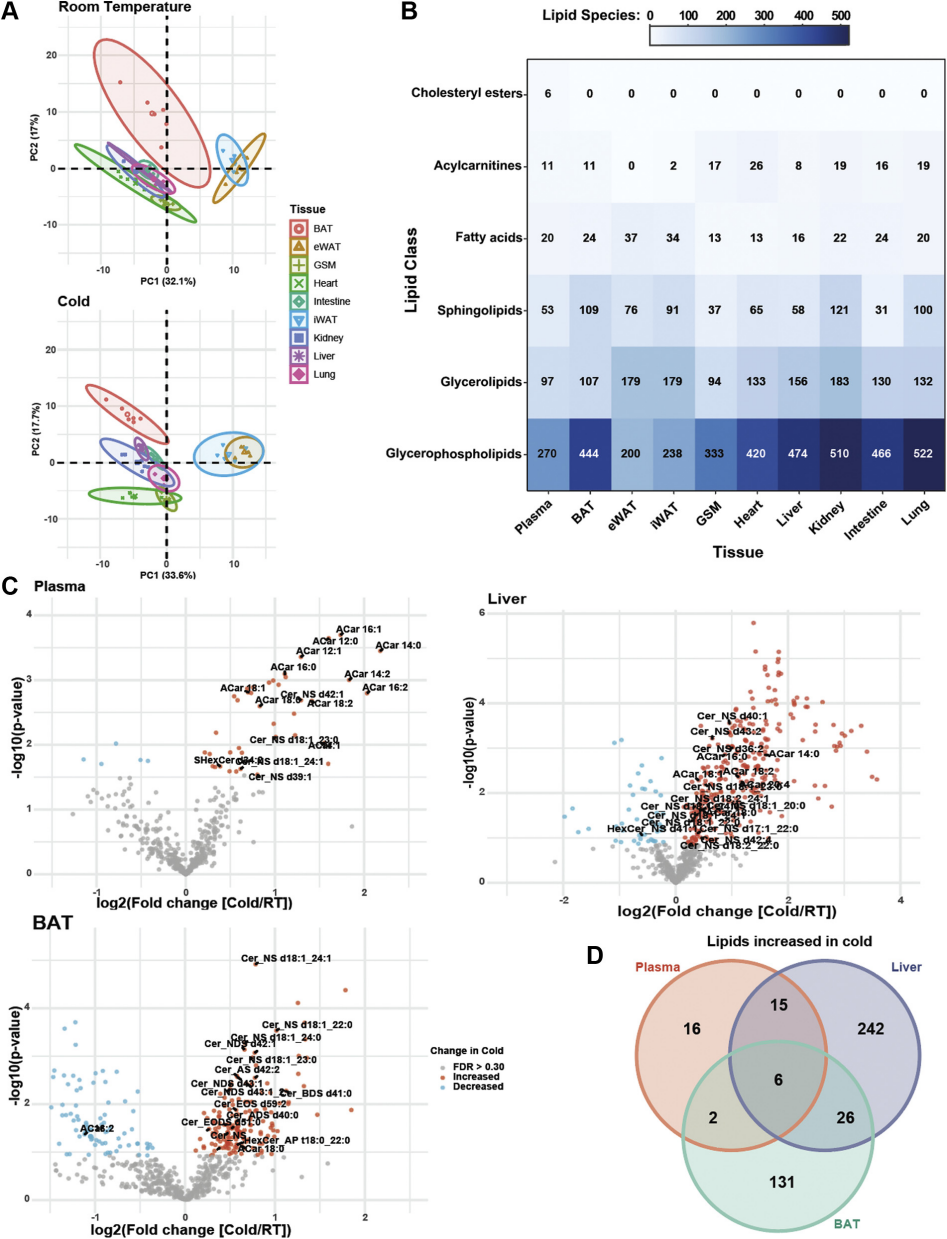
Tissue lipid composition undergoes rapid and dynamic remodeling with cold exposure. A: Principal component analysis between nine tissues in room temperature (24°C) and cold exposure (cold, 4°C). B: Heat map showing lipid class distribution between nine tissues and plasma extracted by IPA method. C: Volcano plots illustrating significant and high fold change between room temperature and cold in plasma, liver, and BAT. D: Venn diagram showing overlap of lipid species significantly increased between room temperature and cold in the plasma, liver, and BAT (*n* = 6 per condition). Student’s *t*-test was used to determine significance in volcano plots with adjustment for multiple comparisons. FDR *q* < 0.30 red dots indicate significant increases in cold and blue dots indicate significant decreases in cold. FDR, false discovery rate.

To identify tissues of origin and processing for the plasma lipid pool, we placed mice in cold (4°C) or control room temperature (24°C) for 6 h while fasting and then harvested 10 tissues for LC/MS lipidomics. Data were normalized to individual ISs for each lipid class and starting tissue amount and was reported semiquantitatively in either nanomolar lipid for plasma or nanomoles lipid/gram for all other tissues. There were 166 lipids commonly detected across all nine solid tissues, and principal component analysis based on these lipids showed clustering by tissue type in room temperature and cold (Fig. 2A). There was a noticeable shift in clustering in cold-exposed mice, but the tissue overlap pattern remained similar. Regardless of temperature, BAT did not overlap with any other tissue, whereas iWAT and eWAT, the main lipid storage depots, clustered together but not with other tissues. For the remaining tissue, there was a high degree of overlap in room temperature, while distinct clustering emerged for GSM and heart in cold exposure.

Next, we looked at the distribution of individual lipid species detected in each tissue for different lipid classes (Fig. 2B). We detected the fewest number of total lipids in plasma (457) and the most in kidney (855). The majority of identified lipids were glycerophospholipids though unsurprisingly, a significant proportion of the WAT depots were composed of glycerolipids. Interestingly, in BAT, we saw more sphingolipid species (109) than glycerolipids (107), which was not observed in any other tissue. There were modest trends in lipid abundances when comparing lipid classes in room temperature and cold for the various tissues (supplemental Fig. S2A). There was a significant increase in liver TGs and FFAs, reflecting the hepatic steatosis induced by cold exposure (12). In both WAT depots, there was an increase in FFAs and diacylglycerols, consistent with increased adipose lipolysis (24). Interestingly, we did not see a significant decrease in WAT TGs associated with lipolysis, which could be due to the normalization by tissue weight rather than fat mass.

To gain insight into which individual lipid species contributed to cold-induced lipid remodeling, we compared the number of lipids significantly changed because of housing temperature after false discovery correction (*q* < 0.30) in each tissue (Figs. 2C, S2B and S1A). We found that lung, intestine, and kidney—the tissues with the most identified lipids—had very few significant changes in lipids during the shift from room temperature to cold (supplemental Fig. S2B). Plasma and liver generally had more lipids increased in cold, driven by increased ACar and Cer species in plasma (Fig. 2C) and increased TG and Cer species in the liver. The most dynamic lipid changes occurred in BAT as there were 165 lipids significantly increased and 107 lipids decreased in cold. The majority of lipids decreased in cold BAT were TGs (82 species), which coincides with increased lipolysis to fuel thermogenesis (29). Unlike plasma or liver, the main lipids increased in cold-exposed BAT were cardiolipin and PC species. Cardiolipins are important lipids for the inner mitochondrial membrane, and their increase is due to higher mitochondrial content in BAT to support thermogenesis (30, 31). In addition, cardiolipins stabilize uncoupling protein 1 structure, support proton transport, and function as signaling molecules inducing thermogenic transcripts (32, 33). While an increase in cardiolipin species has been shown to be more pronounced following prolonged (>3 days) cold exposure, mild increases have been observed after 3 h of cold exposure. There were also significant increases in Cers and ACars in cold BAT.

Because the highest magnitude changes in lipid composition with cold exposure occurred in the plasma, liver, and BAT, we compared the cold-induced lipid changes in the tissue. There were only six lipids commonly increased in all three tissues—ACar 18:0, Cer_NS d18:1_23:0, Cer_NS d18:1_24:1, FA 20:3, FA 22:4, and PC 34:4 (Fig. 2D and supplemental Table S1). There was also more overlap in commonly increased lipid species between plasma and liver (15 lipids) than between plasma and BAT (two lipids). Together, these data indicated a unique lipid profile for each tissue with a subset of tissues exhibiting cold-induced shifts in lipid composition. In particular, the plasma, liver, and BAT had lipid remodeling that was largely distinct from each other but shared some features.

### Tissue of origin and processing for ACars can be predicted using correlation analysis

**Fig. 3:**
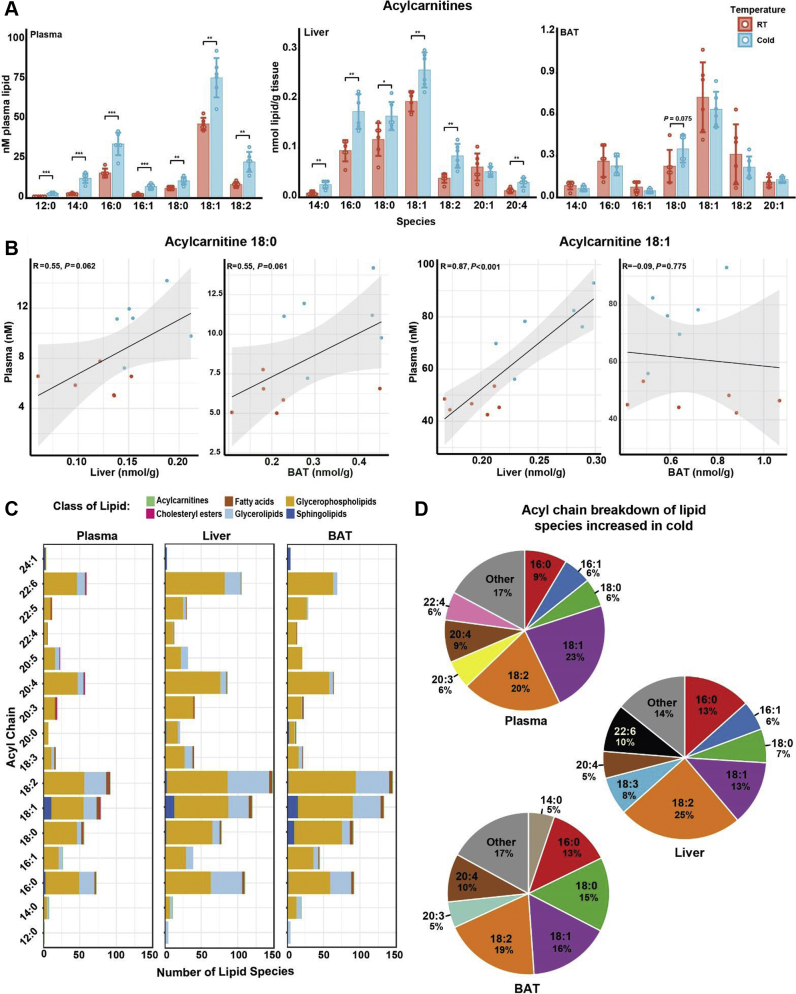
Correlation analysis reveals a plasma, liver, BAT axis for Acar processing. A: ACar species abundance in plasma, liver, and BAT between room temperature (24°C) and cold exposure (cold, 4°C). B: Correlation analysis between plasma, liver, and BAT for ACar 18:0 and ACar 18:1. C: Acyl chain distribution showing the prevalence of different chains across the plasma, liver, and BAT lipidomes. Lipid class occurrence of a given acyl chain is indicated by color. D: Percentage of acyl chain composition of lipids increased in cold from Fig. 2C. Only acyl chains occurring in at least 5% of increased lipids are shown. Student’s *t*-test used for all pairwise comparisons: ∗*P* < 0.05, ∗∗*P* < 0.01, and ∗∗∗*P* < 0.001 (*n* = 6).

One of the shared lipids increased in cold exposure between plasma, liver, and BAT was ACar 18:0. Because of the established relationship of circulating ACars originating from liver and fueling BAT, we compared the most abundant ACar species detected in each tissue during room temperature and cold (9). All major ACar species in plasma and most in liver were increased during cold exposure (Fig. 3A). In the plasma, there were no detected ACars with an acyl chain greater than 18 carbons and no detected ACars with acyl chains below 12 carbons. Although no ACars with acyl chains below 14 carbons were detected in the liver, ACar 12:0 has been shown to significantly increase in the liver after 1 h of cold exposure followed by an increase in serum after 3 h, both of which were maintained through 5 h of exposure (9). This suggested that our current methods may not reliably capture medium-chain (<14 carbon) ACars. In BAT, only ACar 18:0 was increased with cold exposure, but this increase was not significant (Fig. 3A).

To measure the relationship of ACars between the plasma, liver, and BAT, we correlated ACar 18:0, the only ACar increased in all three tissues, and ACar 18:1, the most abundant ACar in all three tissues (Fig. 3B). Plasma ACar 18:0 was positively correlated with both liver and BAT with an identical *R =* 0.55 and *P* = 0.062 and 0.061, respectively. These data were indicative of a general increase in ACar 18:0 in liver that caused an increase in plasma ACar 18:0 and deposited in BAT for thermogenesis (9). In contrast, ACar 18:1 was strongly correlated between plasma and liver (*R* = 0.87; *P* < 0.001) but shared no correlation between plasma and BAT (*R* = −0.09; *P* = 0.775). The absence of correlation was similar across other ACars detected in all three tissues (supplemental Fig. S3A). Upon closer inspection, the lack of significant correlation between plasma and BAT ACars seemed to be driven by room temperature levels (red dots), whereas in cold, BAT ACars trended toward a positive correlation with plasma levels (blue dots).

Our findings recapitulated previously published results demonstrating that cold exposure induces hepatic production of ACars that are secreted into the plasma to fuel thermogenesis in BAT (9). Cold-induced catabolism of ACars could prevent accumulation in BAT. These data suggested that cold exposure could be used to computationally infer the individual lipid contribution of different tissues to the plasma pool. The data also demonstrated acyl chain variation in ACar species between each tissue. We next assessed acyl chain abundance in the various tissues to determine if the presence, or the absence, of specific acyl chains during cold exposure could distinguish between tissues. This information could be used to track the lipid contribution of various solid tissues to the plasma lipid pool.

### Tissue-specific acyl chain signatures are maintained in cold exposure

Under steady-state conditions, each tissue has a distinct acyl chain composition to each lipid class (34). To determine whether tissue-specific acyl chain signatures are maintained in cold exposure, we first compared the number of lipid species containing a given acyl chain in plasma, liver, and BAT (Fig. 3C). General acyl chain distribution patterns were largely similar across all three tissues. The most common acyl chains were 18:2 (first) and 18:1 (second) and existed predominantly in phospholipids, in agreement with previous studies (34, 35). The third most common acyl chain was 16:0 with 73 lipids in the plasma, 110 lipids in the liver, and 92 lipids in the BAT containing this acyl chain. In BAT, 18:0 containing lipids were also common (91 lipids). Upon further inspection, we noticed that this increase in 18:0 containing species was driven by sphingolipids (10 sphingolipids in BAT), which was more than in plasma (one sphingolipid) or liver (two sphingolipids). We next looked at the total intensity of lipids containing various acyl chains and observed the greatest percentage shift in liver and BAT, whereas the total proportions of acyl chain composition remained similar, suggesting that individual lipids species are driving the cold-induced changes (supplemental Fig. S3B).

Next, we compared the major acyl chain distribution (>5%) of lipids increased in cold for plasma, liver, and BAT (Fig. 3D). Similar to general acyl chain prevalence, 18:2 was the most common acyl chain in lipids increased across all three tissues and 18:1 was second. In BAT only, 15% of the acyl chain composition of lipids increased in cold contained 18:0, whereas this number was less than 10% for plasma and liver. These data indicated that an acyl chain signature may differentiate BAT from other tissues. In particular, the presence of sphingolipids containing the 18:0 carbon chain was increased compared with plasma or liver. The 18:0 containing sphingolipids present in BAT were from Cers, and the 18:0 was specifically from the sphingoid base (supplemental Fig. S3C). These Cers were also detected in iWAT and eWAT but not plasma or liver. Total 18:0 Cers were not increased during cold in BAT, but they were significantly increased in iWAT and eWAT. Therefore, 18:0 containing Cers may be lipid signatures for BAT and WAT, an observation that aligns with their known regulation of beige adipocyte differentiation (36, 37). Since these Cers were not detected in the plasma, their functions may be intratissue rather than related to intertissue signaling.

### Regression analysis identifies novel tissue contributions to the plasma ACar pool

**Fig. 4:**
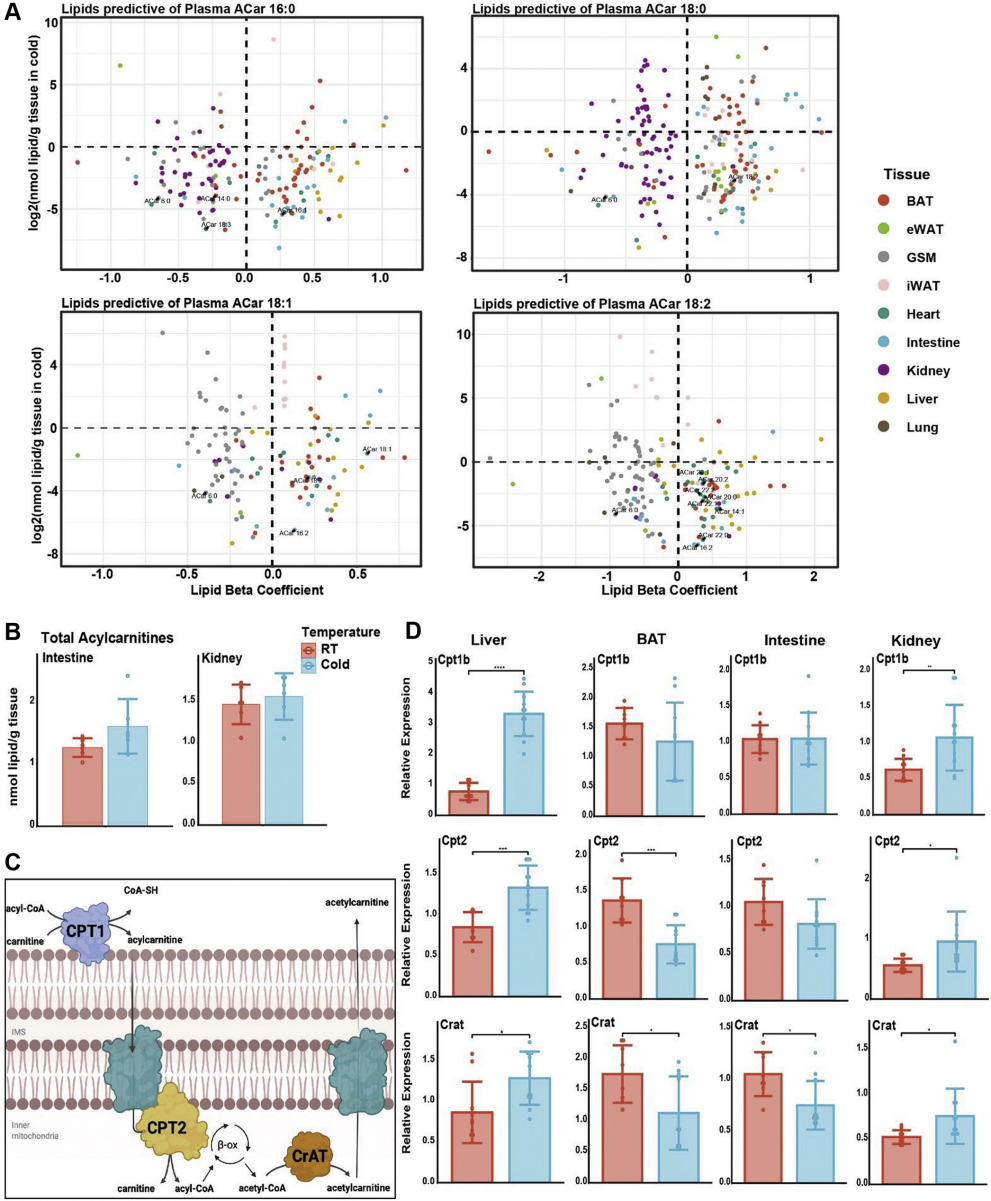
Regression analysis identifies the kidney and intestine as novel regulators of circulating ACars. A: Regression analysis to predict the tissue sources of major plasma ACars. Only lipids significantly predictive (*P* < 0.05) are included. ACars are labeled in black text, and the tissue of origin of lipid predictors is indicated by color. B: Total ACars in intestine and kidney after 6 h at room temperature (24°C) or cold (cold, 4°C). C: ACar metabolism showing key enzymes involved in the pathway. D: RT-PCR analysis of ACar metabolism-related gene expression in several tissues in room temperature versus cold mice. Target gene expression was normalized to the housekeeping gene RPS3. Student’s *t*-test used for all pairwise comparisons: ∗*P* < 0.05, ∗∗*P* < 0.01, ∗∗∗*P* < 0.001, and ∗∗∗∗*P* < 0.0001.

We sought to extend our ACar observations and take an unbiased lipidome-wide approach to potentially identify unknown contributors of the plasma ACar pool. We regressed each lipid identified in our analysis against the most abundant plasma ACars to generate a list of tissue lipids that were significantly predictive of plasma ACars (Fig. 4A). Distinct patterns of lipids from a given tissue were either positive or negative predictors of plasma ACars. Liver and iWAT lipids were generally positively predictive of plasma ACars, which support the known role of these tissues as major contributors of plasma lipids during fasting and cold (9, 24, 38). Most BAT lipids that were significantly changed with cold exposure were also positively predictive of plasma ACars. Since BAT is known to catabolize lipids for thermogenic fuel, the regression analysis may be detecting tissue that contributes to circulating ACars (liver and iWAT) as well as tissues that are major consumers of ACars (BAT).

We observed that intestine ACars were significant predictors of all major plasma ACars (Fig. 4A). This was intriguing as there is growing interest in the intestine as an important contributor of circulating lipids, similar to the liver (39). Total intestine ACars trended higher in cold, but only two species, ACar 16:0 and 20:1, were significantly increased (Figs. 4B and S4A). Though the increase in liver ACars during cold (Fig. 3A) was more widespread than intestine, contribution to the plasma lipidome does not necessitate intratissue lipid changes, and it was possible that the intestine trafficked ACars despite not accumulating them. Numerous kidney lipids were negative predictors of plasma ACars despite no changes in total kidney ACars during cold (Figs. 4A and B, S4A). Accounting for the increased plasma ACars during cold, this may be interpreted as increased kidney ACar uptake and consumption, leaving the kidney ACar pool unperturbed while leading to inhibition of the production of negatively predictive lipids. Alternatively, the kidney may be a contributor to plasma ACars, and the regression results are highlighting a concerted decrease in production, or increase in catabolism, of nonessential lipids to support ACar production and export for the thermogenic program.

Because ACar production is known to be regulated at the transcriptional level, we compared transcriptional changes of genes involved in ACar metabolism between room temperature and cold in the liver, BAT, intestine, and kidney (Fig. 4C and D). Expression of all liver transcripts related to ACar metabolism was significantly increased in the cold, supporting higher production of ACar for export to plasma (Figs. 4C and S4B). BAT uptake and catabolism of circulating ACars was supported by decreases in gene expression of ACar-producing enzymes *Cpt1a* and *Cpt1b*, as well as a decrease in *Crat*, which is known to regulate pyruvate dehydrogenase. This indicates that a decrease in *Crat* could signal the shift from glucose to lipid catabolism (40). Interestingly, *Cpt2*, the enzyme that regulates the carnitine removal in the mitochondria, is increased in the liver and kidney but decreased in the BAT. These observations align with previous findings, including the increases of *Cpt1b* in the liver with cold even though it was originally characterized as a muscle-specific isoform (9, 41, 42). ACar-producing enzymes were not significantly changed in intestine during cold; however, *Crat* expression was decreased. This indicates that while production of intestine ACars is not significantly perturbed in cold, there may be a decrease in mitochondrial export leading to net absorption ACars from circulation by intestine. Interestingly, kidney ACar transcripts had identical patterns to that of the liver. Together with regression results, these data support a role of increased production and contribution of ACars from the kidney into circulation during cold.

Through regression and complementary gene expression analyses, we were able to confirm the liver and BAT as respective contributors and consumers of circulating ACars. We also identified the intestine and kidney as regulators of circulating ACars, a function not previously known for these tissues. These results provide a proof of principle for computational identification of tissue contribution to the plasma pool and warrant the further investigation of ACar metabolism and function in the intestine and kidney during cold exposure.

### BAT and kidney contribute to the circulating Cer pool

**Fig. 5:**
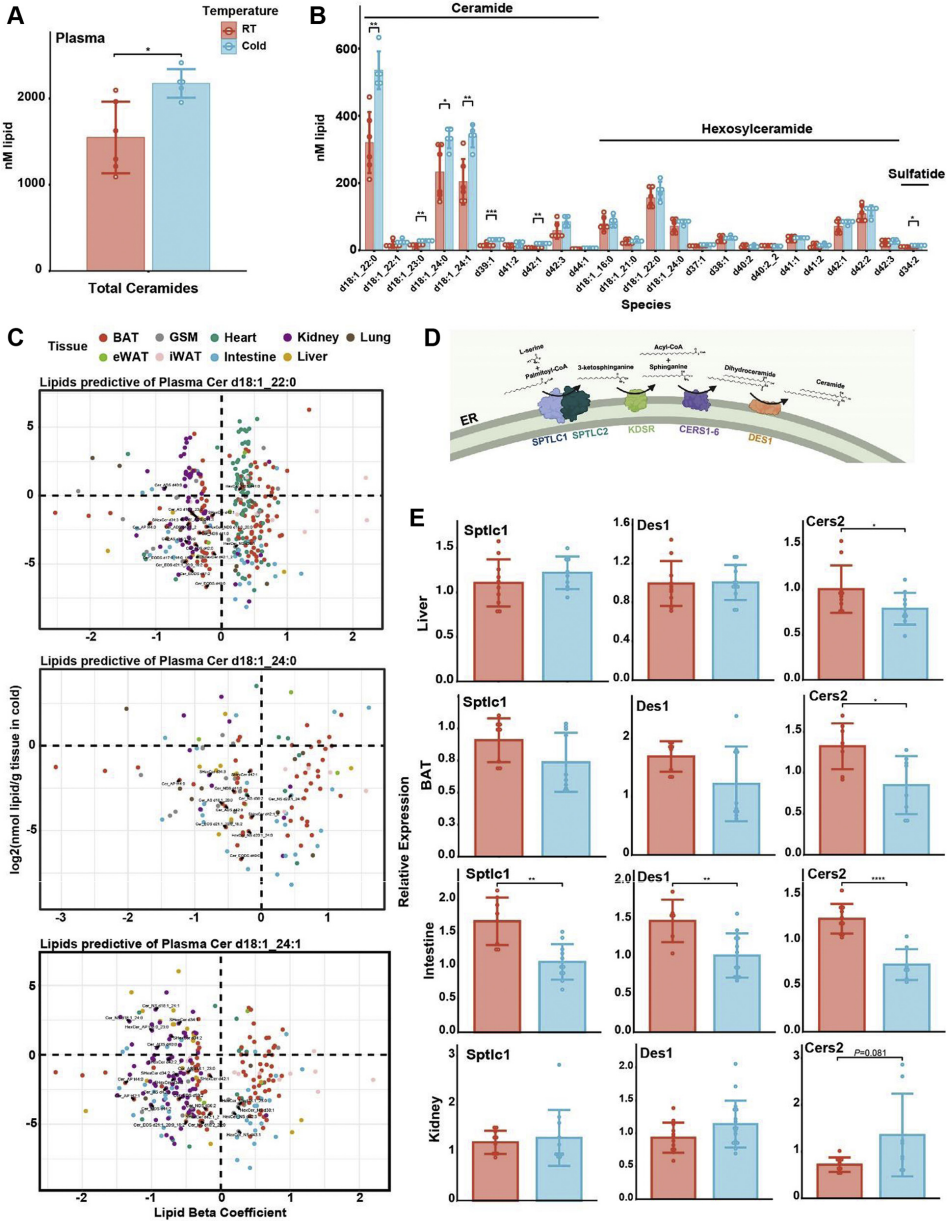
Shifts in Cer levels occur across multiple tissues during cold exposure. A: Total Cer abundance in plasma after 6 h at room temperature (24°C) or cold (cold, 4°C). B: Abundance of all Cer species detected in plasma at room temperature or cold. C: Regression analysis to predict the tissue sources of major plasma Cers. Only lipids significantly predictive (*P* < 0.05) are included. Cers are labeled in black text, and the tissue of origin of lipid predictors is indicated by color. D: Cer synthesis pathway showing key enzymes involved in the pathway. E: RT-PCR analysis of Cer metabolism-related gene expression in several tissues in room temperature versus cold mice. Target gene expression was normalized to the housekeeping gene RPS3. Student’s *t*-test used for all pairwise comparisons: ∗*P* < 0.05, ∗∗*P* < 0.01, ∗∗∗*P* < 0.001, and ∗∗∗∗*P* < 0.0001.

In addition to ACars, Cers, a type of sphingolipid, were the most increased lipids in plasma, liver, and BAT during cold exposure (Fig. 2C). Cers are known to communicate cellular identity as well as regulate insulin signaling and fatty acid oxidation (43, 44). There was a significant increase in total Cers in plasma and liver but not BAT during cold exposure, though the overall level of Cers was higher in BAT than plasma or liver regardless of temperature (Figs. 5A and S5A). When we compared levels of all detected Cer species in room temperature and cold in plasma, we observed that the total Cer increase was primarily driven by Cer d18:1_22:0, Cer d18:1_24:0, and Cer d18:1_24:1 (Figs. 5B and S5B).

To determine which tissue may be contributing to the circulating Cer pool, we performed regression analysis on the major plasma Cers, Cer d18:1_22:0 (C22:0), Cer d18:1_24:0 (C24:0), and Cer d18:1_24:1 (C24:1; Fig. 5C). Lipids from BAT dominated all three regressions as both positive and negative predictors of circulating Cers with BAT Cers generally negatively predictive. This may be indicative of BAT contribution to circulating Cers, though total Cers were unchanged in BAT during cold exposure (supplemental Fig S5A). Liver lipids were poorly represented as significant predictors of circulating Cers despite an increase in total liver Cers during cold (supplemental Fig S5A). This was unexpected given the observation that the liver is the primary regulator of numerous circulating lipids including TGs, ACars, and cholesterol. Intestinal lipids were predictive of all three major circulating Cers, though there was no discernible pattern regarding positive or negative predictive value. We further observed variation in the tissue prediction of circulating Cers based on the Cer species. Many lipids from the heart were positive predictors of C22:0 but not C24:0 or C24:1. Kidney lipids were predominantly negative predictors of circulating C22:0 and C24:1 but not C24:0. Total intestine, heart, and kidney Cers were unchanged in the cold (supplemental Fig. S5A). The range in diversity of Cer species also mirrors these observations with the intestine, plasma, and liver exhibiting similar patterns in Cer species, which could indicate a shared source, whereas the other tissues are more diverse (supplemental Fig. S6A).

We performed gene expression analysis of the major Cer synthesis genes in BAT, liver, intestine, heart, and kidney to further characterize their roles in affecting the circulating Cer pool because transcriptional regulation of Cer synthesis enzymes is indicative of Cer production in prolonged cold exposure (Figs. 5D and E, S6B) (36, 37). BAT expression of the Cer synthesis genes *Cers2*, *Cers5*, and *Cers6* was significantly decreased. Expression of the transcript *Sptlc1*, encoding the first enzyme in Cer synthesis, was also decreased, though not significantly, in the cold. Given that total BAT Cers were unchanged in the cold yet C22:0 and C24:0 were significantly increased (supplemental Fig. S6C), these data support a role of Cer uptake and potentially remodeling in the BAT. A robust Cer program in BAT is further supported by the identification of 70 Cer species—second to only kidney (81 Cers)—many of which trended higher in cold (Fig. 2C).

Heart and liver Cer transcripts were either unchanged or decreased in cold despite an increase in total liver Cers (Figs. 5E, S5A and C). The liver may be a destination for circulating Cers, since the major liver Cers increased were C22:0, C24:0, and C24:1, and there were few liver lipids predictive of circulating Cers. Intestinal Cer transcripts were significantly decreased in cold. This included not only the expression of key synthesis enzymes including *Sptlc1-2*, *Des1*, and several *Cers* but also sphingomyelinases and ceramidases involved in the salvage pathway and remodeling of Cers (Figs. 5E and S5C). These data strongly indicate a broad downregulation of Cer pathways in the intestine, and the associated lack of total Cer changes indicates a role of Cer uptake by the intestine in the cold. In contrast, kidney *Cers2* expression, which controls the incorporation of 22 and 24 carbon chain lengths into Cers, was increased in cold (*P* = 0.081). Though no other kidney Cer transcripts were significantly increased in cold, most synthesis and remodeling transcripts trended higher in cold, and this was a unique feature to kidney. These data indicate that the kidney may play an important role as a contributor to circulating Cers in cold.

The increase in circulating Cers and dynamic changes in BAT Cer species and genes indicates the existence of an important Cer program during thermogenesis. Unlike ACars, the liver does not seem to be a major contributor to circulating Cers but may be a consumer. Our data also implicate the kidney as an important contributor to circulating Cers in cold, whereas the intestine may be involved in uptake. Collectively, these data show a multiorgan Cer program during thermogenesis that results in increased total Cers in the circulation. The function of these Cers remains unknown and is an exciting avenue for further research.

## Discussion

Cold exposure is known to induce maximal lipolysis, shuttling FFAs from stored WAT depots to sites of elevated fatty acid oxidation. We took a systemic approach to understand the impact of this rapid FFA flux on various tissues after acute cold exposure of 6 h. Most tissues were minimally impacted by cold exposure. GSM, intestine, and lung exhibited no significant cold-induced lipid changes, whereas heart and kidney exhibited minor decreases in TGs and increases in ACars. The plasma, liver, and BAT exhibited the most remodeling with cold exposure (Fig. 2C). The two lipid classes that were primarily increased with cold exposure in the plasma were ACars and Cers. Regression analysis indicated that the liver, BAT, intestine, and kidney may have a role in cold remodeling of plasma ACars. Subsequent RT-PCR analysis of ACar production enzymes indicated that the liver and kidney increased production of ACars, whereas the BAT and intestine may take up ACars. Regression analysis also uncovered a multiorgan Cer program in thermogenesis (Fig. 5). In totality, these results demonstrate that lipid composition of tissues is dynamically regulated by physiological stresses such as cold exposure, and computational assessment of these lipids can inform on tissues of production and uptake.

We have previously shown that serum and liver ACars concurrently increase throughout the course of acute cold exposure. Using a ^14^C-ACar 16:0 tracer, the fate of circulating ACars during cold was tracked to catabolism in BAT to fuel thermogenesis (9). In the current study, we recapitulated these findings and extended them by using linear regressions as unbiased computational tools to predict the sources of circulating ACars (Fig. 4A). We were able to confirm the validity of this method by identifying liver and BAT lipids as significant predictors of circulating ACars. However, we had to use complementary gene expression analysis to elucidate the differing roles of liver and BAT as contributors and consumers, respectively, of plasma ACars. These findings highlight the utility and shortcomings of regressions to identify important predictors, but not their function, when considering the relationship between tissue and plasma lipidomes. This is not unique to lipid-lipid analyses but is common in genetic network analyses as well (45). Our regression and RT-PCR analyses also identified the kidney and intestine as potential contributors and consumers, respectively, of circulating ACars. Similar to the liver, the kidney is known to take up circulating lipids in the form of FFA during fasting (46, 47). Interestingly, kidney lipids were only significant predictors for saturated ACars 16:0 and 18:0, but not unsaturated 18:1 or 18:2, and liver ACars correlated better with unsaturated ACar 18:1 than saturated 18:0. This raises the possibility that the liver and kidney contribution to plasma ACars is dependent on ACar species, which may be regulated at the level of substrate availability or transport mechanisms of ACars in the respective tissue. The prediction of the intestine as a source of ACars matches previous studies that show there is a modest increase of intestinal ACars in 24 h fasted rats (48). Together, our results provide novel insight into the role of kidney and intestine as regulators of circulating ACars, but further studies are needed to determine the precise mechanisms and importance of these tissues in lipid-mediated cold adaptation.

We observed a cold-induced rise in several Cers species in the plasma with cold exposure (Fig. 1C). Regression analysis determined that these Cer species may be coming from the BAT and kidney. Several of the most abundant Cer species in circulation were also increased in BAT with cold exposure, and there were no hexosylceramide changes, as others have observed with acute treatment of β-adrenergic receptor agonist (36). Regression analysis also identified the liver as a regulator of plasma Cers and that total liver Cers are increased with cold exposure. The functional role of increased total plasma and liver Cers in acute thermoregulation is unknown. A potential explanation may be related to lipoprotein metabolism. In addition to TG uptake and ACar production, clearance of HDLs is an important function of the liver in cold (49). Still poorly understood, reverse cholesterol transport results in the efflux of cholesterol from peripheral tissue into HDL destined for clearance by the liver. As cholesterol and Cer are known to associate in endosomal pathways, which contribute to cholesterol efflux, a potential explanation for increased liver Cers may be through clearance of HDL particles, which adsorb Cers that are associated with cholesterol (50). Future studies on the mechanisms of Cer transport through circulation should include assessment of exosome and HDL particles, which will inform on the functional role in various physiological stresses.

Cers have been shown to regulate differentiation in brown and beige adipocytes, and adipose tissue-specific knockout of Cer synthesis enzyme *Sptlc2* led to decreased Cers, increased mitochondrial content, increased respiration, and increased thermogenic transcript expression (36, 37, 43, 51). Conversely, loss of Cer degradation through knockout of acid ceramidase 1 driven by the uncoupling protein 1 promoter led to increased Cer levels, decreased mitochondria, decreased respiration, and decreased thermogenic transcripts (37). Modulation of Cer levels in beige adipocytes results in similar metabolic and thermogenic regulation. Acute treatment of beige adipocytes with a sphingolipid synthesis inhibitor, myriocin, increased cellular respiration, whereas increasing Cer levels by treating beige adipocytes with C_2_-Cer led to decreased respiration (36). Beyond regulation of differentiation and respiration, Cer species are known to regulate insulin sensitivity (43, 51, 52). Perhaps in acute cold exposure, where a rapid shift from glucose to lipid catabolism is needed to conserve glucose stores for the brain, Cer-induced insulin resistance confers an adaptive advantage. More work is needed to demonstrate how plasma Cer levels are regulated since expression of Cer synthases decreased in BAT and liver with cold exposure (Fig. 5E), two of the most well-characterized tissues in cold exposure.

One of the major challenges in lipidomics is accurate annotation on global lipidomics sets. The program we used for annotation is LipidAnnotator, which has coverage of 58 lipid types including ether and oxidized lipids using the LipidBlast library (53). While LipidAnnotator provides substantial coverage, its annotation also includes duplications that highlight the need for library curation and further processing to limit the impact on computational assessment (19). We used retention time correlation analysis to determine if duplicate annotations were indicative of true replication, which can occur when combining positive and negative mode, or if they represent spectral overlap of distinct species that cannot be distinguished. Other challenges with annotation in global lipidomics include overannotation, particularly of sphingolipids because of the range and structural similarities numerous species differentiated solely by a double bond or a hydroxyl group. Others have observed similar issues with sphingolipid annotation (54, 55). This highlights the need for global lipidomics to be validated by inspection of matched spectra for independent verification and retention time corrections.

The findings in this article are a systematic assessment of lipid extraction from various tissues, computational imputation of tissue contribution to the plasma lipid pool, and characterization of acute lipid dynamics. We observed that the variability in lipid extraction between methods is different with each tissue, and in the end, we utilized single-phase extraction for ease of protocol and broad coverage of lipid classes between tissues. Application of optimized global lipidomics to each tissue allowed us to determine that acute cold exposure increases plasma ACars and Cers and identify tissues that regulate these circulating pools. These studies are an important step in establishing the functional role of plasma lipids and determining the mechanism through which they are transported into the circulation.

## Data availability

All raw LC/MS data will be deposited in MetaboLights (MTBLS3730). All R code for data processing and curated data for the analysis is available on Github (RJain52:Multi-Tissue-Cold-Exposure-Lipidomics). All other materials are available upon request to the corresponding author.

## Supplemental data

This article contains supplemental data.

## Conflict of interest

The authors declare that they have no conflicts of interest with the contents of this article.
